# 医生在推动中国烟草控制中的作用

**DOI:** 10.3779/j.issn.1009-3419.2010.06.012

**Published:** 2010-06-20

**Authors:** Kylie J. LINDORFF, David J. HILL, 娟 南, 燕 丁

**Affiliations:** 1 Quit Victoria and VicHealth Centre for Tobacco Control, Cancer Council Victoria, Melbourne, Australia; 2 Cancer Council Victoria, Melbourne, Australia and International Union Against Cancer (UICC); 3 天津医科大学总医院，天津市肺癌研究所，天津市肺癌转移与肿瘤微环境重点实验室

**Keywords:** FCTC, 医生, 吸烟, 烟草, 烟草控制

## Abstract

中国已经批准了烟草控制框架公约（Framework Convention on Tobacco Control, FCTC），并致力于执行烟草控制方案，以对抗烟草使用带来的大量不良健康和经济后果。医生需要在这场斗争中像其它国家，如澳大利亚等实施严厉烟草控制政策的国家一样，发挥主导作用。目前中国的问题包括医生自身的吸烟状况及其对患者影响作用的低估，这意味着他们潜在的积极作用远未实现。医生在烟草控制中责无旁贷。首先应从他们自身的戒烟等行为和做法开始，如果医生为吸烟者，则应戒烟，并指导患者及患者家属不吸烟。提倡医院和医疗机构无烟，亦为医生的首要任务。作为人们试图改善自身健康状况和接受疾病治疗的医疗中心，允许吸烟的行为是完全不合理的。

责任亦在于医生工作的机构和代表医生的专业团体机构，应为医生提供有助于控制他们自己以及患者烟草使用的信息、培训和支持。

## 前言

吸烟是全球可预防的导致死亡的最主要原因，而且是全世界八大主要死因中六大死因的危险因素^[1]^。在中国，多达100万30岁以下的男性死于烟草使用^[2]^。作为世界卫生组织（World Health Organisation, WHO）烟草控制框架公约（Framework Convention on Tobacco Control, FCTC）的缔约方，中国致力于执行烟草控制方案，以对抗烟草使用带来的大量不良健康及经济后果。医生可以，而且事实上需要，在这场斗争中发挥主导作用。但是，目前的问题包括医生自身的吸烟状况及其对患者影响作用的低估，这意味着他们潜在的积极作用远未实现。

## 中国的吸烟状况

中国的烟草消费大幅增加，从1970年低于0.5万亿到1990年超过1.5万亿。1990年-2005年间，烟草消费进一步增加至约1.8万亿^[1]^。中国占世界人口的20%，但是在全球约6万亿的烟草产量中，中国消费约占37% ^[3]^。1996年的一次全国调查发现，在中国63%的男性和4%的女性吸烟^[4]^。截止至2002年，这些全国数据并未明显改善，吸烟男性实际上升至66%，吸烟女性略降至3%^[5]^。

这与美国、加拿大、英国和澳大利亚等西方国家的趋势形成对比，这些国家的烟草使用率在近10年显著降低。某些飞速发展中的经济体，如巴西，通过强有力的烟草控制政策和信息传播，吸烟率有所降低^[6]^。这些成绩使人们坚信：鉴于政治承诺和各种资源，中国的吸烟率将会下降。

## 吸烟对健康的影响

吸烟对健康的严重危害是毋庸置疑的。吸烟与肺癌相关的证据出现于20世纪50年代^[7]^，而且在40余年前，肺癌被断定为由吸烟引起^[8]^。吸烟是心脏疾病、中风和呼吸系统疾病的诱因，而且大量的研究显示，吸烟是越来越多的癌症的病因^[9]^。

烟草是肺癌的主要诱因，全球评估显示，烟草引起的肺癌占全部肺癌的90%^[3]^。甚至在澳大利亚等发达国家，肺癌患者的预后亦不良，诊断后仅约11%的男性和14%的女性可存活5年^[9]^。

烟草工业的改革包括过滤嘴的引入，机器测定的焦油量和毒素水平的降低，这些降低了烟草烟雾成分的暴露，但并未降低肺癌死亡率。这些改革可能导致吸烟者癌症类型的改变，与吸烟流行病早期多见的鳞癌相比，目前诊断的癌症类型多为腺癌^[9]^。

尽管与持续吸烟者相比，戒烟者发展成为肺癌的危险性降低，但戒烟者在戒除多年后，其危险性仍较从不吸烟者高^[9]^。吸烟导致肺癌通常需20年左右或更长时间，因此目前肺癌的死亡率反映了20年-30年前公众的吸烟率。

吸烟也是喉癌、口咽癌、食道癌、胰腺癌、胃癌、肾癌与膀胱癌、宫颈癌和急性髓性白细胞病的诱因。亦与肝癌和结肠（肠）癌明显相关。烟草烟雾中有69种已知的致癌物质；它们被吸入肺并通过血流运送至身体其它部位。吸烟也影响代谢与酶的活性，这可能影响致癌作用^[9]^。

## 中国目前及预测的吸烟相关死亡状况

1991年-2000年间，中国男性死亡的主要原因依次为恶性肿瘤、心脏疾病、脑血管疾病、意外事故和感染性疾病。女性死亡的主要原因依次为心脏疾病、脑血管疾病、恶性肿瘤、肺炎和感染以及感染性疾病^[10]^。三大主要死因（心脏疾病、恶性肿瘤和脑血管疾病）占全部死亡病例的66%。吸烟是这三大主要死因的主要危险因素。前五位癌症死因为恶性肺癌、肝癌、胃癌、食管癌和结直肠癌。吸烟可引起或与前五位癌症死因均明显相关。

吸烟引起的死亡率在男性高于女性，城市高于农村，占总死亡率的7.9%，占男性死亡率的12.9%，占女性死亡率的3.1%。有评估认为，如果在中国废除吸烟，男性总死亡率可降低10%，女性总死亡率可降低3.5%^[10]^。

一项全国范围内的代表性研究推算了2005年中国吸烟导致的死亡人数^[11]^。研究显示，2005年，吸烟导致的死亡总人数为673 000例，其中男性538 200例，女性134 800例。三大主要死因为癌症（268 200例死亡）、心血管疾病（146 200例死亡）和呼吸系统疾病（66 800例死亡）。与这些死亡明显相关的特异性疾病为肺癌（129 000例死亡）、中风（20 600例死亡）和慢性阻塞性肺疾病（29 200例死亡）。综合起来，它们约占男性吸烟导致死亡人数的45.1%，约占女性吸烟导致死亡人数的31.8%。这些数据并未考虑被动吸烟导致的死亡人数。

由于中国男性吸烟率较高，因此有人认为被动吸烟在更广泛的公众中亦可明显引起死亡和疾病。被动吸烟可使非吸烟成人罹患心脏疾病和肺癌，并可促使和加剧婴儿、儿童和成人的轻度至重度呼吸系统疾病，增加婴儿猝死综合症和幼童的一系列其它严重健康后果的危险性^[12]^。2006年，美国公共卫生署署长关于二手烟的健康后果的报告中做出推断：没有暴露量就没有危险性^[13]^。Gan等进行了一项旨在评估2002年中国被动吸烟导致成人肺癌的疾病负担的研究^[14]^。他们首次估算了2002年主动吸烟导致肺癌的死亡人数为130 000例，男性114 700例，女性15 300例。随后他们估算了2002年被动吸烟导致肺癌的死亡人数，并发现22 000例肺癌死亡归因于被动吸烟。88%的主动吸烟死亡者为男性，与之不同，女性在被动吸烟中承受着巨大的压力，占被动吸烟死亡人数的80%。22 000例死亡仅为被动吸烟导致肺癌的死亡人数。由于中国有4亿被动吸烟者^[14]^，有人认为与被动吸烟相关的其它疾病和死亡，尤其对于女性和儿童来说，也将增加中国吸烟导致的重大的健康负担。

如果目前的吸烟模式持续下去，中国的死亡人数即可被预测，许多研究已预测了该数字。Peto等预言，目前每年烟草导致的死亡约为1百万，2050年这个数字将会增至约3百万^[15]^。他们声明，如果目前高的吸烟率和低的戒烟率仍不改变，那么在21世纪的前50年中，中国烟草导致死亡的人数将为1亿。

## 经济影响

在中国，吸烟的经济负担是巨大的。据评估，2000年吸烟的经济支出为50亿美元^[16]^。包括治疗吸烟相关疾病的所有卫生保健开支在内的直接支出为17亿美元，占吸烟相关总支出的34%，占2000年中国全民保健支出的3.1%。有研究显示，与发达国家相比，中国处于工作年龄的大多数人群罹患慢性疾病，吸烟对经济的影响具有深远意义^[10]^。

## 中国医生的吸烟率

许多研究评估了中国医生的吸烟率、戒烟方式及态度^[17-20]^。一系列于1987年、1996年和2005年在湖北省省会武汉实施的研究有助于我们监测不同时间段医生间各指标的变化^[18, 20]^。男医生的吸烟率在1987年-1996年间从50.9%显著升高至61.3%，2005年略降至58.0%。女医生的数据更令人担忧。1987年女医生的吸烟率仅为4.8%，1996年升至3倍达12.2%，2005年再次增加超过一半达18.8%。

各项研究均比较了医生的吸烟状况与其参与戒烟指导的可能性的关系。在1987年，医生的吸烟状况与他们是否参与戒烟指导显著相关，非吸烟医生明显更易于参与对患者的指导。然而在1996年，事实并非如此，医生的吸烟状况与其参与戒烟指导的可能性无关^[20]^。在2005年，非吸烟医生明显更易于参与戒烟指导，70.5%的非吸烟医生经常或总是劝导其患者戒烟，但仅48.6%的吸烟医生参与戒烟指导^[18]^。

2004年，一项仍然在湖北省实施的研究调查了农村医生的吸烟状况，为城市医生与农村医生间的差异提供了一些见解^[19]^。研究发现，农村男医生的吸烟率为31.9%，如按性别进行分类，农村女医生均为非吸烟者。吸烟率也随着年龄而变化，低于25岁的年轻人吸烟率最低（6.3%），50岁-54岁人群的吸烟率最高（31.6%）。但是，鉴于这项研究是在教学医院实施的，尤其是由于年轻的医学生在医院不允许吸烟，因此相应的数据可能偏低^[18]^。

吸烟状况、戒烟指导行为和态度的差异不仅存在于农村医生和城市医生之间，而且存在于不同城市的医生之间。2004年一项基于6个中国城市的医生的医院调查发现，男性吸烟率为41%，女性吸烟率为1%^[17]^。这些数据明显低于湖北省武汉市医生的吸烟率。2008年一项源自977家医院大约40 000名医生的近期研究发现，男性吸烟率为38.7%，女性吸烟率为1.1%，但是不同地区间吸烟率不同^[21]^。当对像中国这样庞大人群的国家的医生实施戒烟支持、培训或教育时，辨识和理解区域间的差异的意义是成功的关键所在。

不同科室医生的吸烟率亦不同，比如外科医生、妇科医生、儿科医生、中医医生、整形外科医生和重症监护室医生^[18, 19]^。当执行基于医生的烟草控制计划时，需要考虑医生的专业方向。

## 医生的吸烟状况对烟草控制成功的影响

有关中国医生吸烟状况数据的持续搜集尤为重要，因为它是象征目前烟草控制方案有效性的“晴雨表”。医生作为健康的行为榜样，如果持续吸烟，就不易于说服公众戒烟。其次，有研究显示，一个国家烟草流行的“成熟度”可通过医生的吸烟率来预测，因为他们往往比一般人群更早戒烟^[22]^。

澳大利亚医生吸烟率的下降为解释该现象提供了一项实用的病例研究。在20世纪60年代，大约1/3的澳大利亚医生吸烟，总人口的吸烟率也较高，超过1/3的女性吸烟，接近2/3的男性吸烟^[23]^。在20世纪70年代中至晚期，医生的吸烟率几乎减少了一半，而且在19世纪80年代早期，仅约1/10的医生吸烟。在20世纪90年代，医生的吸烟率甚至进一步降至1/20。尽管在20世纪90年代，一般人群中男性吸烟率也有所降低，但与医生吸烟率的下降不一致，一般男性吸烟率约为1/4。女性吸烟率在20世纪60年代至20世纪80年代起初有所升高，但在20世纪90年代降至约1/5^[23]^。

强有力的证据显示，医生的干预作用严重影响其患者戒烟的可能性^[24]^。来自澳大利亚等国家的证据也表明，伴随着越来越多的医生戒烟，他们更多地参与其患者的戒烟指导。在1964年，不到一半的澳大利亚医生指导其患者戒烟，但到1982年，91%的医生参与其患者戒烟^[23]^。因此，当医生作为行为榜样在健康行为方面起到更好的模范带头作用时，他们也更易于鼓励患者不吸烟或戒烟。

医生是影响患者的可信的和权威的资源。而且，在中国，许多医生似乎并未在健康行为的榜样方面和鼓励其患者戒烟方面发挥应有的重要作用。

据报道，37%的吸烟医生在其患者面前吸烟^[17]^，51%的吸烟医生很少或从未进行戒烟指导^[18]^。在所有的医生中，21.1%的医生对他们应该为其患者树立不吸烟的榜样有异议，仅21.4%的医生认为医生是帮助患者成功戒烟的最有影响力的人^[18]^。

## 哪些是需要做的？

解决逐渐增多的烟草相关疾病的难题应依赖更好的治疗还是更好的预防？尽管将最新研究转化为更行之有效的治疗是至关重要的，但权威专家赞成更多地关注预防。世界卫生组织秘书长Margaret Chan声明：治愈这一致命疾病并非依赖药物或疫苗，而是依赖政府和公众社会的一致行动^[1]^。为此，我们还应强调：医生需要负起责任并发挥带头作用。

中国的研究显示，与医生自身的吸烟状况一样，有多种因素与医生戒烟指导的频率显著相关。包括他们对责任的认知、对成功的认知、对模范作用的认知以及他们是否坚信他们在劝阻患者戒烟中具有强大的影响力^[18]^。鼓励和支持医生自己戒烟的教育和计划是提高他们参与患者戒烟指导的第一步。为医生提供关于短暂干预戒烟的培训可产生双重功效，为鼓励医生自己戒烟提供信息和动力，并为其为患者解决这一问题时提供技巧和信心。

医生亦可影响吸烟行为及体制和组织层面的政策。他们可以利用他们作为受尊敬的专家及社会活动家的职务提倡经证实的烟草控制方案。提倡医院和医疗机构无烟应为医生的首要任务。作为人们试图改善自身健康状况和接受疾病治疗的医疗中心，允许吸烟的行为是完全不合理的。

在中国解决吸烟的责任亦在于医生工作的机构和代表医生的专业团体机构。医院和医疗机构的管理者和经营者应该执行无烟环境、确保检查和记录、张贴政策，以明示所有的吸烟患者并为医生提供他们戒烟所需的必要支持，比如提供持续的教育和培训。代表医生的专业团体亦应是使其成员发生改变的载体，告知医生最新关于烟草相关危害的研究，鼓励他们成为积极的公众模范且不吸烟，而且利用他们作为专业机构的权威以提倡政府的基于循证的烟草控制干预。

## 哪些支持是可以采纳的？

幸运的是，对医生、医疗卫生机构、决策制定者和政府的大量指导有助于其执行基于循证的烟草控制方案。WHO FCTC是拥有超过165个缔约方的多边条约，旨在对抗全球的烟草流行^[25]^。FCTC为各国降低烟草供应和烟草需求规划了一幅蓝图。中国批准履行了FCTC，并因此承诺通过共同抗击烟草来保护中国公民的健康。

在FCTC下，许多指南已被采纳，有助于各缔约方履行其义务。他们提供了最佳的现有证据及已经成功执行烟草控制方案的其它缔约方的经验。政府采纳的指南是关于：防止烟草控制方面的公共卫生政策受烟草行业的商业和其它既得利益的影响（公约第5.3条）；防止接触烟草烟雾（公约第8条）；烟草制品的包装和标签（公约第11条）和烟草广告、促销和赞助（公约第13条）^[26]^。

关于教育、交流、培训和公众意识（公约第12条）和与烟草依赖和戒烟有关的降低烟草需求（公约第14条）的指南也正在制定中。

2008年WHO全球烟草流行报告：MPOWER系列政策包含了6项重要的烟草控制政策，这为执行FCTC提供了进一步的指导^[27]^。2008年报告首次全面地分析了全球烟草使用状况和烟草控制的努力状况。2009年公布的第2版MPOWER报告关注了无烟政策的执行情况^[28]^。报告显示，中国仍有待提供无烟的卫生保健机构。

2009年5月，中国卫生部及其它相关部委印发了题为“关于2011年起全国医疗卫生系统全面禁烟的决定”的政策。决定提出了确保2011年实现卫生行政部门和医疗卫生机构全面禁烟的策略和方案的具体工作计划^[29]^。在全国实现无烟示范医院的进程取得了缓慢进展，这将为其它国家的学习提供借鉴和培训场所^[30]^。为了进一步协助医生，在2009年年底临床医生戒烟指南已被修订并重新发布。该指南旨在协助医生明示吸烟者、诊断和治疗烟草依赖以及提供及时的戒烟服务^[31]^。在推进中国快速实现卫生部规定的无烟政策及履行FCTC规定的义务中，医生均发挥着巨大作用。

## 总结

治疗烟草相关疾病的医生，尤其是治疗肺癌的医生，在烟草控制中责无旁贷。首先应从他们自身的行为和做法开始，如果医生为吸烟者，则应戒烟，如果其继续吸烟，这提供了一个信息：医生可能并不相信吸烟有害健康。积极开展关于肺癌患者戒烟的咨询是当地社区传播信息的有效途径。努力实现医疗场所的无烟环境。了解烟草控制机构开展的烟草宣传活动，如果可能的话予以支持。

医生对中国烟草控制的成功至关重要。他们积极参与解决烟草使用过度的难题，有助于预防许许多多可避免的过早死亡。

## Acknowledgments

Thank you to Marina Haritos, Lin Li and Indra Haslam from the Cancer Council Victoria, Australia, for research assistance with this paper.
